# The Complexity of Treatments and the Multidisciplinary Team—A Rare Case of Long-Term Progression—Free Survival in Prostate Cancer until Development of Liver and Brain Metastases

**DOI:** 10.3390/jcm12175579

**Published:** 2023-08-27

**Authors:** Roxana-Andreea Rahnea-Nita, Laura-Florentina Rebegea, Alexandru Nechifor, Cristian Mareș, Radu-Valeriu Toma, Alexandru-Rares Stoian, Anda-Natalia Ciuhu, Liliana-Florina Andronache, Georgiana Bianca Constantin, Gabriela Rahnea-Nita

**Affiliations:** 1 The Clinical Department, The Faculty of Medicine, The University of Medicine and Pharmacy “Carol Davila”, 050474 Bucharest, Romania; roxana_rahnea@yahoo.com (R.-A.R.-N.); dr.marescristian@gmail.com (C.M.); tomaraduvaleriu@yahoo.com (R.-V.T.); dr.raresstoian@yahoo.com (A.-R.S.); andronache.liliana@yahoo.com (L.-F.A.); gabriela_rahnea@yahoo.com (G.R.-N.); 2 The Oncology-Palliative Care Department, “Sf. Luca” Chronic Disease Hospital, 041915 Bucharest, Romania; andadum@yahoo.com; 3 The Radiotherapy Department, “Sf. Ap. Andrei” County Emergency Clinical Hospital, 800579 Galati, Romania; laura_rebegea@yahoo.com; 4 The Clinical Department, The Faculty of Medicine and Pharmacy, “Dunarea de Jos” University in Galati, 800008 Galati, Romania; alexandru.nechifor@ugal.ro; 5 The Research Center in the Field of Medical and Pharmaceutical Sciences, ReFORM-UDJ, 800010 Galati, Romania; 6 The Urology Department, “Sf. Ioan” Emergency Clinical Hospital, 042122 Bucharest, Romania; 7 The Radiotherapy Department, The Oncological Institute “Prof. Dr. Alexandru Trestioreanu”, 022328 Bucharest, Romania; 8 The Surgery Department, “Bagdasar-Arseni” Emergency Clinical Hospital, 041915 Bucharest, Romania; 9 The Morphological and Functional Sciences Department, The Faculty of Medicine and Pharmacy, “Dunarea de Jos” University in Galati, 800008 Galati, Romania

**Keywords:** prostate cancer, liver and brain metastases, long-term survival

## Abstract

Introduction: Prostate cancer has no initial clinical manifestation in the case of brain metastases since they are asymptomatic at first. This is why there is a high risk for clinicians to overlook these lesions, and they are often confused with other diseases. With all the improvements in diagnostic technological methods, which allow the early detection of lesions, and the progress in terms of systemic therapy associated with increased survival, an increase in incidence has also been noticed. Materials and methods: We report the case of a 64-year-old patient who presented himself to the Oncology Department of “St. Luca” Chronic Disease Hospital in Bucharest in November 2011 and received the following diagnosis: biopsied prostate neoplasm, local-regionally advanced, pelvic lymph node metastases. Results: After receiving complex oncological treatment, this patient represents a rare case of long-term progression-free survival (15 years). Discussions: This case has some particularities. According to the literature data, survival with metastatic prostate cancer is approximately 21 months, and cerebral metastases are found in only 2% of prostate cancer cases. This case is one of the few cases in the specialty literature that benefited from all therapeutic sequences; namely, total androgenic blockade, docetaxel, abiraterone, enzalutamide, and cabazitaxel. Conclusions: Brain metastases are an unfavorable prognostic factor in prostate cancer. The therapeutic options developed in recent years allow the improvement of survival.

## 1. Introduction

Prostate cancer is the second most frequent cancer among men worldwide and the fifth leading neoplastic cause of death [1]. The main risk factors involved are advanced age, family history, and African-American race, but current research also highlights lifestyle-related factors, such as diet, a sedentary lifestyle, and obesity [2]. 

Clinically, presentation ranges from a localized asymptomatic form within a low-risk group disease to aggressive disease with distance dissemination. Approximately 17% of prostate cancers lead to metastases, the most frequent location being the bony skeleton (approximately 85%), followed by the lymph nodes, liver, and lungs (approximately 10%) [3,4].

Cerebral dissemination related to prostate cancer is a negative prognostic sign and it occurs with a very low frequency in approximately 2% of the cases [5].

Given the fact that there is no initial clinical manifestation in the case of brain metastases due to prostate cancer (since they are asymptomatic at first), there is a high risk for clinicians to overlook these lesions. They are often confused with abscesses, meningiomas, or subdural hematomas in CT scans and it is not uncommon for them to be detected only at necropsy [6].

With the advancement of diagnostic technological methods, which allow the early detection of the lesions, and progress in terms of systemic therapy associated with increased survival (the occurrence of secondary determinants in the CNS being late), an increase in incidence has been noticed [7,8].

Patients are usually excluded from clinical trials and there is no established systemic or intrathecal therapy. Docetaxel hardly crosses the hemato-encephalic barrier, and for Cabazitaxel, although it can better cross it, no clear benefit has been established in terms of its effectiveness [9,10]. The second-generation antiandrogens, such as enzalutamide, are considered to be related to a high risk of convulsive seizures [11]. Abiraterone, a synthetic androgen inhibitor, crosses the hemato-encephalic barrier, but its role in the antineoplastic activity at this level is uncertain [12].

Taking into account the information above and due to the fact that it is a rare condition, at present there is no consensus regarding the therapeutic conduct for brain metastases in prostate cancer, so a review of each diagnosed case and collection of data about the evolution of these patients according to the therapy they were approached with are important for a better understanding that could lead to determining an optimal strategy.

## 2. Specialty Literature

By performing a review of the specialty literature, we collected some interesting data on the topic, considering that we had a case for which we wanted to observe similarities and differences compared to it.

A very interesting study conducted research by collecting all the articles related to the topic of brain metastases in prostate cancer. It analyzed 27 articles that appeared in the specialty literature between 1976 and 2021, highlighting certain characteristic elements [5].

The following was of interest to our observations. Increased incidence was noticed among a younger population (the average age was 59 years) with castration-resistant prostate cancer (CRPC), metastases predominantly at the dural level, and an average Gleason score of 9 [13].

However, on the other hand, after the analysis of the 27 articles, it was noticed that there was no connection between the increased incidence due to imaging progress and that due to increased survival through progress in systemic therapy, considering that there were no significant differences between the incidence among naive patients and those resistant to castration [14].

Brain metastases related to prostate cancer were located in the brain parenchyma, dura mater, or leptomeninx, and an interesting fact was that cancers with an intraparenchymal location were associated with the best prognosis, unlike cancers from other locations, where brain metastases located in the dura mater showed a better survival rate [15].

Other significant aspects were the fact that brain metastases were found with a higher frequency in relation to histopathological types other than adenocarcinoma, such as neuroendocrine or small cell carcinoma, this being a possible predictive factor for brain metastases. Moreover, lung and liver metastases were found to be independent risk factors [5].

In terms of treatment, an improvement in the survival rate was noticed for patients whose systemic treatment was changed, given the progression of the disease under previous therapy, and for whom local therapy, such as radiotherapy or resection, was considered to be beneficial, especially in patients with single metastases [5,16]. 

As previously mentioned in the introduction, there is no consensus regarding the treatment of choice, with prognosis being unfavorable, and overall survival has not undergone significant improvements in recent years [5].

## 3. Clinical Case Presentation

The introduction and the review of data from the specialty literature aimed to introduce the presentation of a case of prostate cancer with brain metastases, which was a special case not only due to this aspect but also due to long-term survival (2008–2023), both with a local-regional disease (2008–2019) and a metastatic one (2019–2023). Such long-term survival led to the involvement of a multidisciplinary team (a urologist, an oncologist, a radiotherapist, a neurologist, an ophthalmologist, and a palliative care physician), the patient benefiting from almost all treatment options available for prostate cancer up to the end of his life, when palliative interventions were performed to improve his life quality [17].

The case concerned a patient who was 64 years old at the onset of the disease (March 2008) and presented himself to the Oncology Department of “St. Luca” Chronic Disease Hospital in Bucharest in November 2011, receiving the following diagnosis: biopsied prostate neoplasm, local-regionally advanced, pelvic lymph node metastases. Computed tomography (CT) of the thorax and magnetic resonance imaging (MRI) of the pelvis showed structural and dimensional prostatic modification, with a tumor aspect affecting the medial lobe with limited extension at the level of the seminal vesicles, isolated pelvic lymph node metastases (T3b, N1, M0-IVA staging), and a prostate-specific antigen (PSA) level of 3.4 ng/mL.

The histopathologic examination performed in March 2008 revealed prostate carcinoma with a Gleason score of 6 (3 + 3), the patient thus falling into the low-risk group.

Previously, the patient underwent hormonal treatment with bicalutamid in another medical facility.

The patient was fully active, with an Eastern Cooperative Oncology Group (ECOG) performance status = 0.

In November 2011, a total androgen blockade was initiated represented by the first-generation antiandrogen bicalutamid + LH-Rh analog. This treatment was employed for 8 years with various combinations that were changed periodically, with the administration of LH-Rh analog modified at various intervals between 2011 and 2019: monthly (3.75 mg triptorelinum), every 3 months (11.25 mg triptorelinum or 10.8 mg goserelinum), and every 6 months (45 mg leuprorelinum). Throughout this period, the follow-up CT indicated stationary disease with PSA normalization. 

The computed tomography performed in June 2013 revealed a medial prostatic proliferation, with slight infiltration of the seminal vesicles on the midline and the postero-inferior vesical wall; subdiaphragmatic adenopathies; focal lesions at the level of the right hepatic lobe with an angiomatous loading, most likely hemangiomas (the largest of the cavernomatous type); mild adrenal hyperplasia; right renal serous cysts; and a small gastric hiatal hernia. 

**Figure 1:**
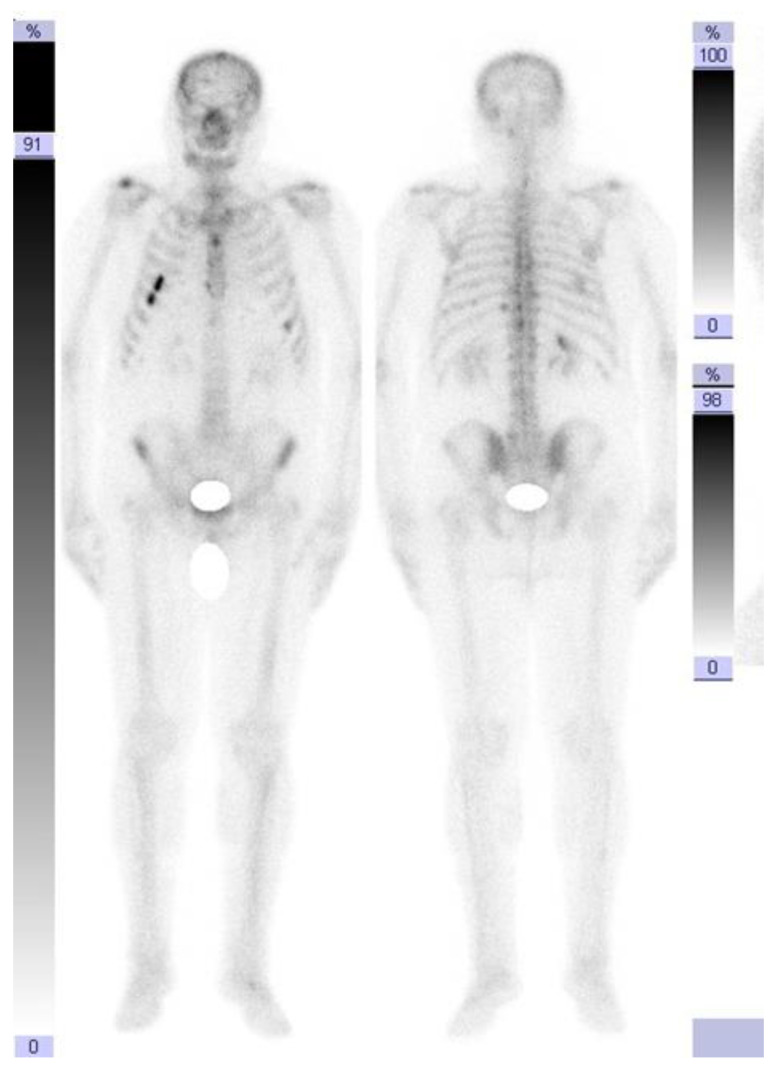
Bone scan (September 2019).

However, in September 2019, the follow-up computed tomography indicated the need to perform bone scans for newly appearing suspicious lesions at the costal level, which revealed left costal osteogenic reactions that had recently appeared (Figure 1).

Small hypercapture foci located at the level of the left antero-lateral costal rib cage CVI, chondro-costal CVII, and posterior left CVII, CVII, and CIX—the changes had various intensities and dimensions—represented the metabolically active aspect.

An increase in PSA (30 ng/mL), which had been normal throughout this period, was also noticed. Therefore, we decided to initiate chemotherapy with Docetaxel for metastatic disease and also treatment with osteoclast inhibitors for bone metastases. 

After seven series performed with 21-day intervals, a new tomography revealed a post-biopsied prostate neoplasm and dimensional progression in the prostate with invasion in the left seminal vesicle. No secondary pulmonary or hepatic determinations or mediastinal or abdominal-pelvic tumor adenopathies were noted. There was progression of some osteocondensations on the rib cage. 

Treatment with abiraterone (an androgen biosynthesis inhibitor) + prednisone + Lh-Rh analog was initiated, a treatment indicated for castration-resistant prostate cancer, which progressed after the docetaxel-based therapy. After 9 months of treatment, in April 2021, the PSA value was 66 ng/mL, and a new imaging evaluation through computed tomography revealed a prostate with increased dimensions (axial diameters: 48/45 mm) and the presence of a medial lobe and a diffusely non-homogenous structure. The seminal vesicles presented no significant densitometry particularities. The liver had a non-homogenous structure, with the presence of a hypocapturing mass with axial diameters of 28/26 mm located at the subscapular region at the border of segments VI and VII, suspected from the CT to be a secondary-type lesion. 

**Figure 2:**
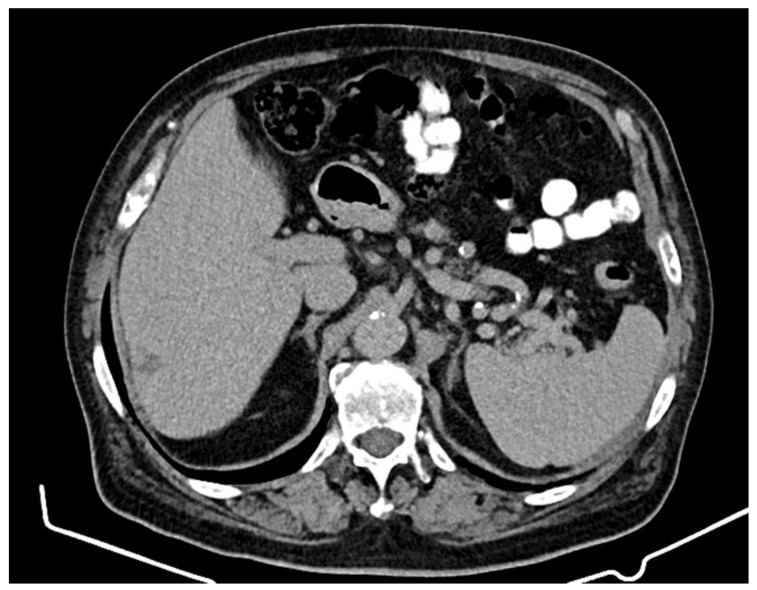
Computed tomography (April 2021): liver with non-homogenous structure, with the presence of a hypocapturing mass with axial diameters of 28/26 mm. Middle and lower interaorticocaval and lumboaortic retroperitoneal lymph nodes with maximal axial diameters of 17/11 mm.

**Figure 3:**
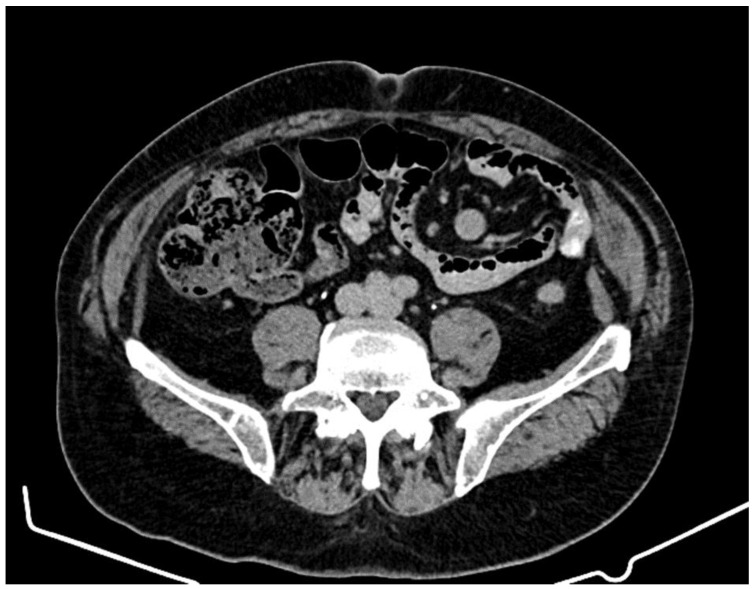
Computed tomography (April 2021): prostate with increased dimensions (axial diameters of 48/45 mm). The seminal vesicles presented no significant densitometry particularities.

The presence of intra-aortocaval and middle lower lumboaortic retroperitoneal lymph nodes with maximum axial diameters of 17/11 mm was observed. There were no ascites at the abdominal-pelvic level. No inguinal adenopathy or hydro-aerial distensions in the intestinal abdominal-pelvic area were observed. Multiple focal osteocondensed, sternocostal, clavicular, scapular, and thoraco-lumbar vertebral lesions were observed, as well as lesions at the level of the bony pelvis with an appearance suggestive of secondary bone determinations; practically, this represented a significant progression of secondary bone determinations (Figure 2 and Figure 3).

Treatment with a second-generation antiandrogen—namely, enzalutamide + zoledronic acid—was initiated, which the patient underwent for 9 more months (May 2021–January 2022), throughout which he initially had a favorable response with a PSA decrease (30 ng/mL). Pain caused by bone metastases was treated with nonsteroidal anti-inflammatory drugs and weak opioids, depending on its intensity (low/moderate). The computed tomography of the head, chest, abdomen, and pelvis in September 2021 revealed a dimensional reduction in the prostate compared to the previous examination in April 2021. A stationary hypodense liver lesion dimensionally comparable to the previous examination was observed, along with adenopathy of approximately 1.3 cm in the aortopulmonary window.

**Figure 4:**
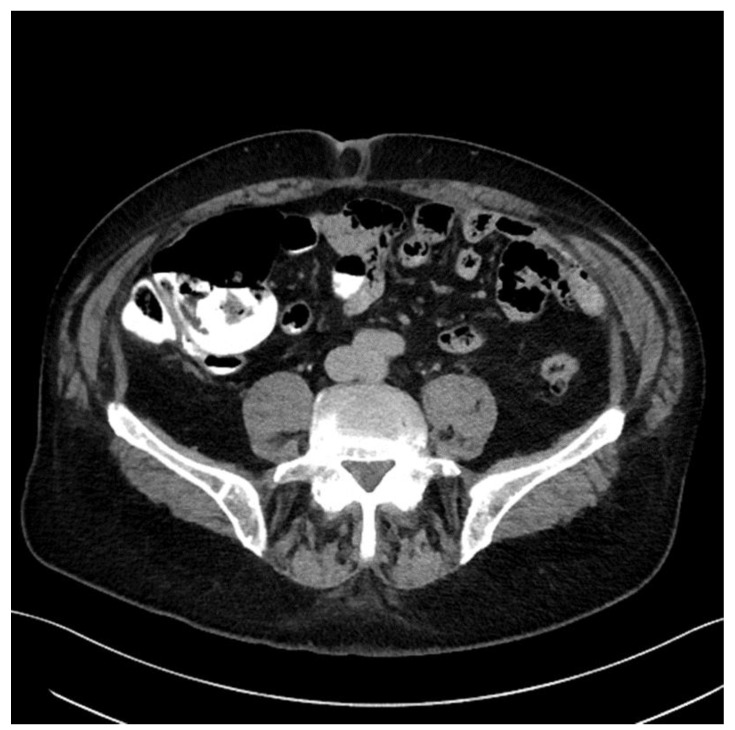
Computed tomography (September 2021).

Numerical and dimensional reductions in the lymph node metastases at the abdominal-pelvic level were observed, along with osteocondensed lesions at the level of the scanned bone segments. No lesions suggestive of secondary determinations at the level of the brain or lung parenchyma were noted (Figure 4). 

A dimensional reduction in the prostate compared to the previous examination in April 2021 was observed, along with a hypodense hepatic lesion stationary in dimensions compared to the previous examination. There was an adenopathy of approximately 1.3 cm in the aortopulmonary window.

Numerical and dimensional reductions in adenopathies at the abdominal-pelvic level were observed, along with osteocondensed lesions at the level of the scanned bone segments.

**Figure 5:**
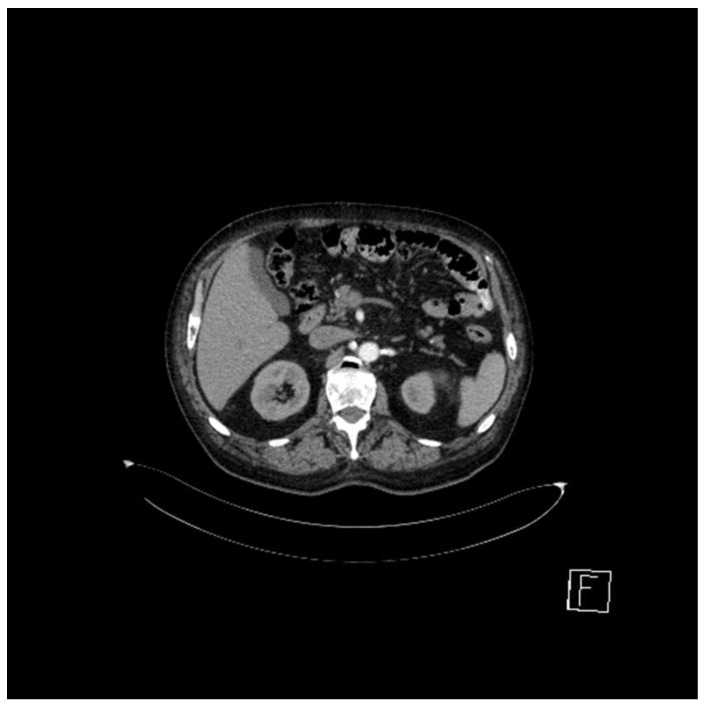
Computed tomography (January 2022): hypocapturing hepatic lesions, two of them with a stationary appearance compared to September 2021 and one not visualized upon the previous examination. For additional characterization. an abdominal MRI with intravenous contrast medium was recommended. No new secondary brain or pulmonary determinations were observed. Secondary osteocondensed bone determinations, some of them with slight dimensional progression, were noted.

Subsequently, the evolution was unfavorable, and computed tomography of the head, thorax, abdomen, and pelvis performed in January 2022 revealed hypocapturing hepatic lesions, one of them not visualized during the examination in September 2021, and secondary osteocondensed bone determinations, some of which showed slight dimensional progression (Figure 5). 

Cabazitaxel was then initiated (January 2022), a drug indicated for metastatic prostate cancer in patients previously treated with docetaxel. The treatment was undergone for 6 months, a period in which the PSA changed from 70 ng/mL to 1000 ng/mL, associated with the result of the abdominal MRI performed in August 2022, which showed two liver lesions suspicious for secondary determinations and multiple bone secondary determinations. 

The treatment with cabazitaxel was interrupted and the overall hormonal treatment was initiated with an antiandrogen + Lh-Rh analog, taking into account the deterioration in the Eastern Cooperative Oncology Group (ECOG) performance status from 0 to 2–3.

A lung X-ray was performed in October 2022, which revealed multiple nodular opacities, and cerebral computed tomography was recommended due to the newly occurring symptomatology involving visual disturbances, nausea, vomiting, and dizziness and a PSA over 1000 ng/mL. The CT revealed a sequelar subdural hematoma versus a right-sided hygroma and multiple cerebral secondary determinations.

This was the moment when the question about oncological treatment by means of radiotherapy for brain metastases versus best supportive care arose [17].

The multidisciplinary board decided to perform radiotherapy, aiming to improve the patient’s life quality. He underwent external “whole brain” radiotherapy with a Varian accelerator by means of the 3D conformational technique in October 2022, with DT = 30 Gy/10fr/12 days and a dose/fraction = 3GY/fraction, showing good tolerance to treatment and improvement in the neurologic symptoms. He continued the hormonal treatment for another two months, which was associated with palliative symptomatic treatment represented by hepatoprotective, cerebral antidepletive, and anticonvulsant treatment, as well as antialgic treatment with mild opioids, hydration IV lines, and psychological counseling, until the time of death, which occurred at the end of January 2023 [18].

This work referred to a patient who had a healthy and balanced diet, was a non-smoker, and did not consume alcohol. At the same time, the patient was an optimistic person; he was very calm, had great trust in the medical team, and was also a religious person. He wrote down all the aspects related to his illness in a diary and communicated them to the doctor at each consultation. He also strictly followed all medical recommendations. It was a privilege to treat such a patient.

## 4. Discussion

1. The overall survival with metastatic prostate cancer is approximately 21 months [19]. Depending on the histopathologic type, the risk group, and the PSA levels, more than 50% of locally advanced cases at diagnosis progress to metastatic disease, despite the treatment administered [20]. Taking into account these data from the specialty literature, the particularity of the case presented in this paper was represented by the inconsistency between the aggressiveness of the disease (stage IV A–T3b, N1, M0) with distant metastases and the fact that the patient was in a low-risk group, with a Gleason score of 6 and a PSA level slightly above the upper limit of normal at the time of diagnosis.

2. The therapeutic treatment of choice for locally advanced castration-resistant disease is the androgenic blockade (antiandrogen + Lh-Rh analog), aiming to prolong progression-free survival (PFS). Although most patients develop castration-resistant disease, another particularity of the case appearing here was the fact that PFS was maintained after a period of 11 years from the time of diagnosis.

3. Moreover, another particularity of the case consisted in the occurrence of cerebral metastases, a situation found with a frequency of only 2% [5].

A question mark arises related to the fact that standard conduct regarding the staging and monitoring of locally advanced prostate cancer patients does not involve brain imaging in the absence of neurological symptoms [21,22].

In the case of our patient, two brain scans were performed before the onset of symptoms, both in 2021 and at the beginning of 2022, which did not reveal any secondary determinations. 

4. Regarding the therapeutic sequences, this was one of the few cases in the specialty literature that benefited from all therapeutic sequences: total androgenic blockade, docetaxel, abiraterone, enzalutamide, and cabazitaxel. According to the specialty data, fewer than 15% of patients get to benefit from the third line of treatment.

5. Towards the end of the patient’s life, the question of deciding between cerebral radiotherapy and the continuation of the oncologic treatment versus best supportive care arose. The multidisciplinary team chose the first option.

Probably, the decision to continue or not continue treatment for patients at the limit of indication with a moderately impaired performance status is one of the most difficult decisions that clinicians have to deal with. It is perhaps the situation in which the risk–benefit balance arises most strongly. In the present case, it was proven to be beneficial to the patient as it palliated the neurologic symptoms and improved his quality of life [17]. Due to multiple metastatic sites (bone, liver, lung, lymph nodes), the impaired performance status, and the choice of the patient and his family, stereotactic brain biopsy was not an option.

6. Last but not least, the atypical nature of the case, consisting in the extremely long survival of 15 years (2008–2023), should have been at the top of the discussion.

## 5. Conclusions

Metastatic prostate cancer remains a worldwide problem with an impact on the life quality of the population, given the increased number of patients diagnosed with advanced disease, a situation in which a very high percentage reach distant dissemination.

The most frequent location for metastasis is the bony skeleton, followed by the lungs, the liver, and, more rarely (2%), the brain. The introduction of brain imaging as a monitoring investigation method, and not only in the presence of specific symptoms, could be beneficial for the early detection of dissemination at this level.

Brain metastases are an unfavorable prognostic factor, the overall survival in the specialty literature being 2.5 months [23].

The therapeutic options have developed in recent years, thus leading to improved overall survival [24]. In addition to the first-generation hormonal therapy, new classes of drugs have appeared, such as second-generation antiandrogens (enzalutamide) and androgen biosynthesis inhibitors (abiraterone), and, in addition to the option of docetaxel, cabazitaxel has also appeared.

This aspect was also reflected in the case presented in the paper: overall survival of 15 years with locally advanced prostate cancer at diagnosis and survival of 4 years with metastatic disease. 

It is important that research is continued in order to establish a new consensus regarding the optimal therapeutic sequences, which are still not clearly defined [25]. Palliative care once again demonstrated its beneficial role, improving our patient’s life quality at the end of his life.

## Data Availability

No new data were created or analyzed in this study. Data sharing is not applicable to this article.
